# 

**Published:** 1848-02

**Authors:** 


FEBRUARY AND MARCH, 1848.

VOL. II. NEW SERIES. NO. VI.

Luther Jewett

### ILLINOIS AND INDIANA

### MEDICAL AND SURGICAL

### JOURNAL.

PUBLISHED EVERY OTHER MONTH.

EACH NUMBER CONTAINING NINETY-SIX PAGES.

EDITED BY

JAMES V. Z. BLANEY, M. D., DANIEL BRAINARD, M. D.,
WM. B. HERRICK, M. D., AND JOHN EVANS, M. D.

PROFESSORS IN RUSH MEDICAL COLLEGE, CHICAGO, ILLINOIS.

CHICAGO, ILL.:
PUBLISHED BY WILLIAM ELLIS.

INDIANAPOLIS, IND.:
PUBLISHED BY JOHN D. DEFREES.

1848.

TWO DOLLARS PER ANNUM, IN ADVANCE.

